# Neuropsychological profile and correlation with outcomes in patients admitted to spdc

**DOI:** 10.1192/j.eurpsy.2021.373

**Published:** 2021-08-13

**Authors:** M. Benassi, F. Ambrosini, R. Raggini, G. Piraccini, R. Sant’Angelo

**Affiliations:** 1 Department Of Psychology, University of Bologna, Cesena, Italy; 2 Psychologist, Independent Researcher, Savignano, Italy; 3 Mental Health Department, AUSL ROMAGNA, Cesena, Italy

**Keywords:** Neuropsychological profile, cognitive functions, problem solving, outcomes

## Abstract

**Introduction:**

Literature showed that patients suffering from disorders belonging to the schizophrenic (SZ) and bipolar (DB) spectrum have a qualitatively similar but quantitatively different neurocognitive impairment that correlates with the outcomes. However, the majority of former studies are conducted on patients in remission phase.

**Objectives:**

This study aims to compare cognitive functions between SZ and DB in the acute phase and their possible correlations with treatment outcomes.

**Methods:**

In a prospective longitudinal study conducted at the SPDC Ausl unit of Romagna - Cesena, 57 SZ and 82 DB took part in the study. The diagnosis was based on the SCID5 CV and SCID5 DP. Symptom severity was assessed with BPRS and HONOS both at the beginning and at the end of hospitalization. Executive functions were measured with Tower of London (ToL) and Modified Wisconsin Card Sorting Test (MCST), attention with Attentive Matrices (MA) and Stroop Test (ST), non-verbal logic skills with Colored Matrices by Raven (PM47). The statistical analyzes applied are ANOVA and logistic regression.

**Results:**

The cognitive tests did not reveal significant differences between SZ and DB. The logistic regression analysis showed that the scores obtained at the MCST and MA positively correlate with the efficacy of the treatment for both groups.

**Conclusions:**

Cognition in DB and SZ patients was similarly impaired, supporting recent theories that placed diagnoses on a continuum of severity. Moreover, the results indicated that also in the acute phase the best predictors of the outcome were flexibility in problem solving strategies and visuospatial attention.

**Disclosure:**

No significant relationships.

